# Detection and quantitation of host cell proteins in monoclonal antibody drug products using automated sample preparation and data-independent acquisition LC-MS/MS

**DOI:** 10.1016/j.jpha.2021.05.002

**Published:** 2021-05-20

**Authors:** Lisa Strasser, Giorgio Oliviero, Craig Jakes, Izabela Zaborowska, Patrick Floris, Meire Ribeiro da Silva, Florian Füssl, Sara Carillo, Jonathan Bones

**Affiliations:** a Characterization and Comparability Laboratory, NIBRT–National Institute for Bioprocessing Research and Training, Dublin, A94 X099, Ireland; b School of Chemical and Bioprocess Engineering, University College Dublin, Dublin, D04 V1W8, Ireland

**Keywords:** Data-independent acquisition, Host cell proteins, Critical quality attributes, Liquid chromatography-mass spectrometry, Monoclonal antibody, Chinese hamster ovary cells

## Abstract

Ensuring the removal of host cell proteins (HCPs) during downstream processing of recombinant proteins such as monoclonal antibodies (mAbs) remains a challenge. Since residual HCPs might affect product stability or safety, constant monitoring is required to demonstrate their removal to be below the regulatory accepted level of 100 ng/mg. The current standard analytical approach for this procedure is based on ELISA; however, this approach only measures the overall HCP content. Therefore, the use of orthogonal methods, such as liquid chromatography-mass spectrometry (LC-MS), has been established, as it facilitates the quantitation of total HCPs as well as the identification and quantitation of the individual HCPs present. In the present study, a workflow for HCP detection and quantitation using an automated magnetic bead-based sample preparation, in combination with a data-independent acquisition (DIA) LC-MS analysis, was established. Employing the same instrumental setup commonly used for peptide mapping analysis of mAbs allows for its quick and easy implementation into pre-existing workflows, avoiding the need for dedicated instrumentation or personnel. Thereby, quantitation of HCPs over a broad dynamic range was enabled to allow monitoring of problematic HCPs or to track changes upon altered bioprocessing conditions.

## Highlights

• Reproducible HCP analysis using automated, magnetic bead-based sample preparation.
• Quick and easy implementation into pre-existing LC-MS peptide mapping workflows.
• DIA-LC-MS/MS for comprehensive analysis of low abundant HCPs, contaminating peptides without additional sample pretreatment.

## Graphical abstract

**Figure:**
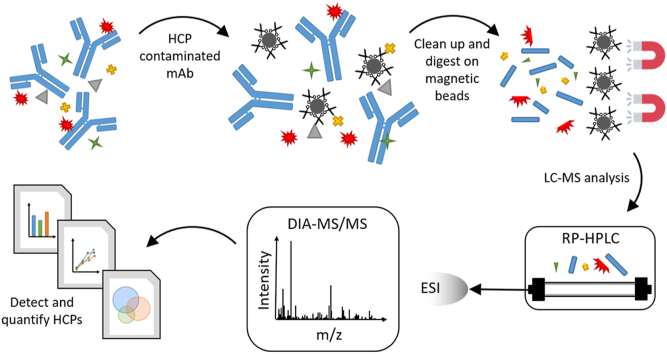

## 1 Introduction

Monoclonal antibodies (mAbs), which are commonly produced in Chinese hamster ovary (CHO) cells, constitute the most significant part of biopharmaceutical drug production. In recent years, mAb production processes have been steadily optimized to achieve higher productivity and efficiency [1]. Contaminating host cell proteins (HCPs), however, are a recurrent problem that requires consistent monitoring [2]. In particular, the presence of degrading or immunogenic proteins, such as cathepsins or phospholipase B-like 2 (PLBL2), have been shown to affect product quality even at trace levels and thus need to be analyzed [3].

The current standard approach to quantifying HCPs is based on enzyme-linked immunosorbent assay (ELISA), which measures the overall amount of HCPs present. Thus, orthogonal methods, such as liquid chromatography-mass spectrometry (LC-MS), are required to detect and quantify specific contaminants [4]. Recently, a multiple reaction monitoring (MRM)-based LC-MS workflow has been reported, allowing for a robust targeted quantitation of PLBL2 [5]. Nevertheless, one substantial challenge encountered by untargeted LC-MS-based approaches is the dynamic range due to the low abundance of HCPs compared with a high concentration of mAb present in the drug substance or drug product. Traditionally employed data-dependent acquisition (DDA) might not sufficiently cover the required dynamic range to allow for adequate detection or quantitation of HCPs. One commonly applied approach to circumventing this problem is to resolve co-eluting peptides by pre-fractionation [[6], [7], [8]]. Another possibility is to reduce the dynamic range using molecular weight cutoff (MWCO) filters or by targeted depletion of the mAb prior to analysis [9,10]. Recently, alternative digestion approaches have been employed [11,12]. These techniques have been successfully used to monitor HCP contamination in various CHO-derived biopharmaceutical products. However, owing to their higher complexity, these methods tend to be time-consuming and error-prone, which makes them difficult to implement in the biopharmaceutical industry. A promising alternative to DDA, which has been proven to be very powerful in recent years, is data-independent acquisition (DIA). With DIA it is possible to cover a higher dynamic range, and since fragment spectra for all eluting peptides are acquired irrespective of their abundance, it allows for a more comprehensive analysis without depending on prior fractionation [13,14].

This work aimed to establish a simplified DIA-based workflow for sensitive HCP analysis, which can easily be implemented using the same instrumental setup routinely used for standard peptide mapping analysis of mAbs [15]. Additionally, the use of a commercially available, magnetic bead-based automated sample preparation enables high throughput and reproducibility. The suitability of the presented method was tested by analyzing the effects of applied changes in bioprocessing parameters on the HCP profile of a CHO DP-12-derived anti-IL8-IgG1 antibody solution after protein A purification. The presented workflow is easy to implement and represents a way to combine various characteristics such as automation, reproducibility and robustness, as well as deep HCP coverage in an unprecedented manner. Therefore, it is ideally suited for implementation in the biopharmaceutical industry.

## 2 Materials and methods

### 2.1 Materials

Purified water was obtained from an Arium Pro UV ultrapure water system (Sartorius Stedim Biotech, Goettingen, Germany). LC-MS grade water containing 0.1% (*V/V*) formic acid (FA) and LC-MS grade acetonitrile containing 0.1% (*V/V*) FA were obtained from Fisher Scientific (Dublin, Ireland). All reagents and materials required for digestion such as Thermo Scientific™ SMART Digest™ trypsin kit, Thermo Scientific™ KingFisher Deepwell 96-well plates, and 12-tip combs were obtained from Thermo Fisher Scientific (Sunnyvale, CA, USA). DL-dithiothreitol (DTT) (≥98%), iodoacetamide (IAA), Trizma® base, phosphate-buffered saline (PBS), and glycine were purchased from Sigma-Aldrich (Wicklow, Ireland). Hi3 Phos B standard for label-free Hi3 quantitation of detected HCPs was obtained from Waters (Wexford, Ireland). A commercially available IgG1, rituximab, was provided by the Pharmacy Unit of the University Hospital of San Cecilio in Granada, Spain.

### 2.2 CHO DP-12 cell culture

Experiments using an anti-IL8-IgG1-producing CHO DP-12 cell line (clone #1934, aIL8.92 NB 28605/14, ATCC® CRL12445™) were performed in batch cultures using 3 L Finesse SmartGlass vessels (Thermo Fisher Scientific, Waltham, MA, USA). Cells were grown in BalanCD CHO Growth A media (FUJIFILM Irvine Scientific, Wicklow, Ireland) containing 4.0 mM L-glutamine (Sigma-Aldrich, St. Louis, MO, USA). Bioreactors were inoculated with 4 × 10^5^ cells/mL. For standard conditions, the pH and temperature of the cultures were maintained at 7.00 ± 0.05, and 37 °C, respectively, and dissolved oxygen (DO) concentration was maintained at 40% of air saturation. For suitability testing, the processing conditions were altered on day 6. Apart from standard conditions, cells were also grown at a lower temperature of 32 °C or at a DO level lowered to 20% or a combination of these using single replicates.

### 2.3 Protein A affinity chromatography

Cells were harvested on day 10 and the supernatants were clarified via centrifugation and sequential filtration through 0.45 μm and 0.20 μm filters (VWR, Dublin, Ireland). Purification of the expressed mAb was performed using an ÄKTA avant 150 chromatography system with a 1 mL HiTrap Protein A HP column (GE Healthcare, Dublin, Ireland). The sample was loaded at a flow rate of 1 mL/min, followed by a wash step with 20 mM sodium phosphate (pH 7.0) and the elution of the IgG with 50 mM sodium citrate at pH 3.0. The eluate was neutralized with 1.0 M Tris and then buffer-exchanged to PBS using Vivaspin® 2 3 kDa MWCO spin filters (Sartorius Stedim Biotech, Goettingen, Germany). The samples were assayed for IgG content using a 4 mm × 35 mm Thermo Scientific™ MAbPac™ Protein A column (Thermo Fisher Scientific, Sunnyvale, CA, USA). A 10 μL aliquot was loaded onto the column at a flow rate of 0.5 mL/min using 50 mM sodium phosphate and 60 mM NaCl (pH 7.5, buffer A). After a 1 min isocratic hold at 100% A, the sample was eluted using 50 mM sodium phosphate and 60 mM NaCl at pH 2.5 (buffer B) for 3.5 min. Subsequently, the column was re-equilibrated with buffer A for 5 min. The absorbance of the resulting solution was measured at 280 nm, and the protein concentration was evaluated after performing a calibration curve using an IgG1 standard (data not shown). 0.10, 0.25, 0.50, 1.00, and 2.00 μg of rituximab were loaded onto the column.

### 2.4 Sample preparation for LC-MS analysis

Tryptic digestion was performed using Thermo Scientific™ SMART Digest™ trypsin kit and the Thermo Scientific™ KingFisher Duo Prime system following the manufacturer's protocol and as previously described [15]. Briefly, 100 μg of the sample (2 mg/mL) was diluted with 150 μL of SMART digest buffer in a Thermo Scientific™ KingFisher Deepwell 96-well plate. Following a wash step, magnetic beads obtained from 15 μL of magnetic SMART bead solution were added to the sample and incubated at 70 °C for 60 min. Following digestion, disulfide bonds were reduced by incubating the samples in 10 mM DTT for 20 min at room temperature, followed by alkylation with 3 mM IAA at room temperature for 15 min in the dark. Subsequently, the samples were evaporated to dryness and resuspended in 0.1% FA in H_2_O to a final concentration of 1 μg/μL.

### 2.5 Reversed-phase (RP)-LC-MS/MS for peptide mapping and HCP analysis

Peptide mapping of the IgG1 samples was performed as reported previously [15], using a Thermo Scientific™ Vanquish™ Flex UHPLC system (Thermo Fisher Scientific, Germering, Germany) coupled to a Thermo Scientific™ Q Exactive™ Plus Hybrid Quadrupole-Orbitrap™ mass spectrometer using a Thermo Scientific Ion Max™ API source equipped with a heated electrospray ionization (HESI-II) probe (Thermo Fisher Scientific, Bremen, Germany). For peptide mapping analysis, 8 μg of tryptic peptides were injected onto a Thermo Scientific™ Acclaim™ VANQUISH™ C_18_ UHPLC column (250 mm × 2.1 mm, 2.2 μm; Thermo Fisher Scientific, Sunnyvale, CA, USA), and 100 μg of the sample were loaded for HCP analysis using the same experimental setup. Briefly, the separation of tryptic peptides was performed using a linear gradient of 2%–40% acetonitrile containing 0.1% (*V/V*) FA for 70 min at a flow rate of 0.30 mL/min and a column temperature of 25 °C. The mass spectrometer was operated in positive ion mode and a spray voltage of 3.8 kV was applied. The capillary temperature was 300 °C and the sheath gas flow was set to 40 arbitrary units. For HCP analysis, data were acquired using variable isolation windows in the DIA mode. Full scans were acquired for a scan range of *m/z* 150–2000 using a resolution setting of 70,000 (at *m/z*  200) and an AGC target of 1e6 with a maximum injection time of 200 ms. DIA-based fragment scans were acquired for a scan range of *m/z* 210–1410 using a resolution setting of 17,500, an AGC target of 1e6 and a normalized collision energy of 28 eV. The isolation windows were set to *m/z* 40.0, 20.0, and 10.0, respectively. To enable accurate label-free quantification, Hi3 Phos B standard was added to the samples to obtain a final injection amount of 3 pmol. All samples were analyzed in triplicate.

### 2.6 Data analysis

Raw data were processed using Progenesis QI version 2.2, (Nonlinear Dynamics, Newcastle, UK) enabling match-between-runs (using default settings) and including charge states between +2 and +5. The ion intensity maps generated were exported as a pep.Xml file for protein identification and quantification using Thermo Scientific™ Proteome Discoverer™ software version 2.1. A database search was performed using Sequest™ HT against a *Cricetulus griseus* database (UP000001075 downloaded from UniProt on April 20, 2018) appended with the sequences of the Hi3 protein standard and the anti-IL8-IgG1 sequence. Search criteria allowed a maximum of two missed cleavages, a mass tolerance of 10 ppm for the precursor ions and 0.8 Da for the fragment ions, carbamidomethylation of cysteine as a static modification, oxidation of methionine as a variable modification and a false discovery rate of 1%. The resulting mgf files were processed in Progenesis QI for relative quantitation using the Hi3 standard. Raw data were deposited onto the ProteomeXchange Consortium via the PRIDE partner repository [16] with the dataset identifier PXD020127.

## 3 Results and discussion

### 3.1 Peptide mapping

**Fig. 1:**
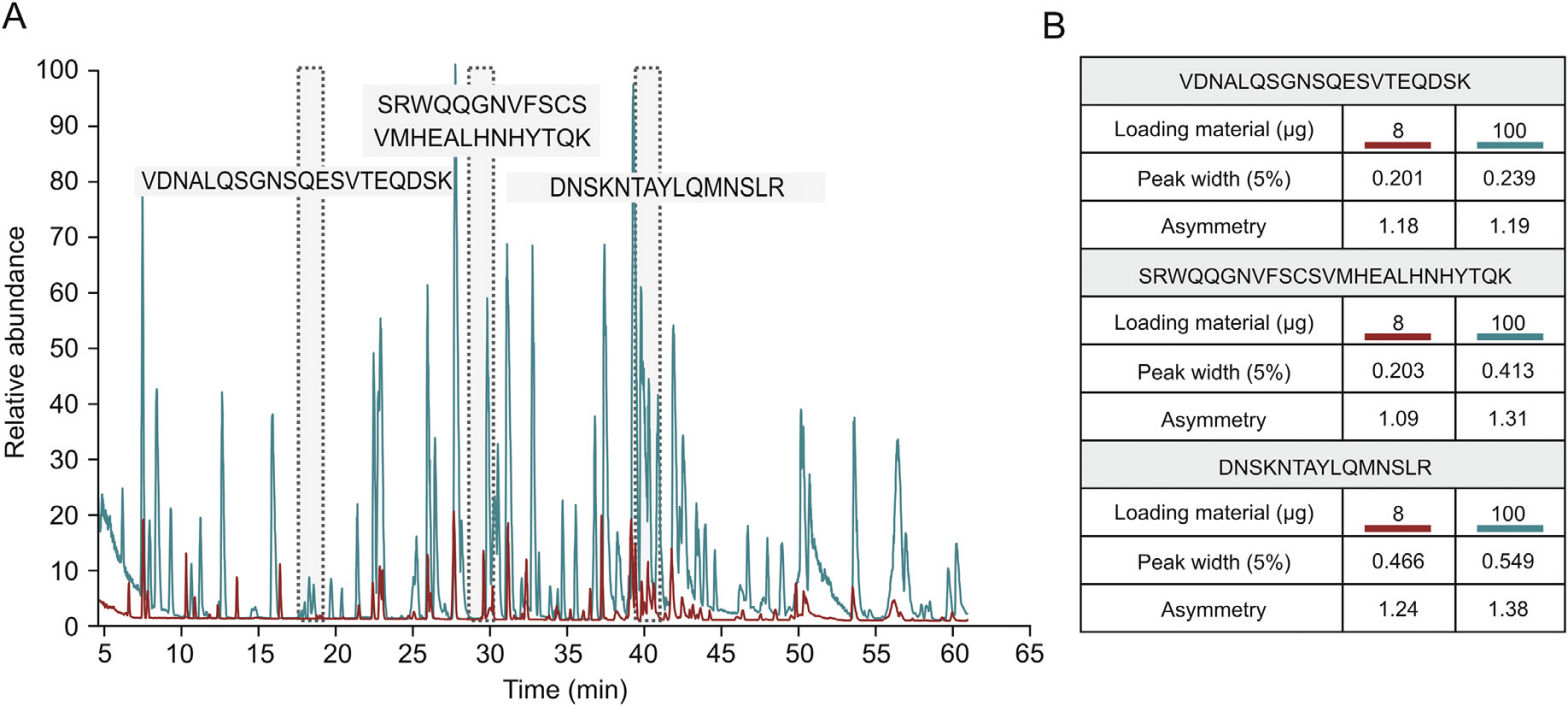
Peptide map of CHO DP-12-derived anti-IL8-IgG1 after protein A purification. (A) Base peak chromatograms comparing the standard loading amount for peptide mapping (8 μg, red) and the higher loading amount for HCP detection (100 μg, blue). Highlighted in gray are the IgG1-derived peptides used to assess chromatographic performance. (B) Comparison of peak width and asymmetry of selected peptides when loading 8 μg vs. 100 μg of a sample onto the analytical column.

Peptide mapping is a standard analytical method used by the biopharmaceutical industry to assess the critical quality attributes (CQAs) of biological products. Peptide mapping is used to confirm the identity of a drug substance by providing information on its amino acid sequence, and it also provides site-specific information about post-translational modifications (PTMs), such as oxidation or deamidation, which can require close monitoring [17]. This study aimed to establish a method for HCP monitoring that can easily be implemented in standard peptide mapping workflows using the same instrumental setup for LC-MS analysis. Additionally, the use of an automated sample preparation workflow reduces the required processing time and yields excellent reproducibility [18]. However, to improve HCP coverage, an injection amount higher than that usually used for the standard peptide mapping of a drug substance was applied for HCP analysis. While 8 μg was used for peptide mapping, 100 μg was injected onto the column for HCP analysis. In both cases, 100% sequence coverage of the mAb under investigation was obtained (data not shown). Since the chromatographic performance is related to the sample amount injected, an evaluation was performed to assess the impact of the 12.5-fold increase in the amount of material on the column on the quality of the separation obtained. Based on the extracted ion chromatograms of three peptides chosen for evaluation due to their difference in elution time, their peak width and asymmetry were compared when injecting 8 or 100 μg (Fig. 1A). As shown in Fig. 1B, peak asymmetry remained constant, while as expected, the peak width was slightly increased with higher injection amounts, showing that chromatographic performance was largely maintained.

### 3.2 HCP detection and quantitation

In recent years, multiple studies have employed DDA-based LC-MS/MS approaches to detect and quantify HCP impurities in several different mAb products. While these studies presented sensitive and reproducible orthogonal methods when compared to ELISA [2], most protocols require some form of sample pre-treatment to improve HCP detection [6,9,10]. However, the industry is looking for sample preparation and analysis procedures that are simple, reproducible, robust, and easy to implement in existing workflows.

**Fig. 2:**
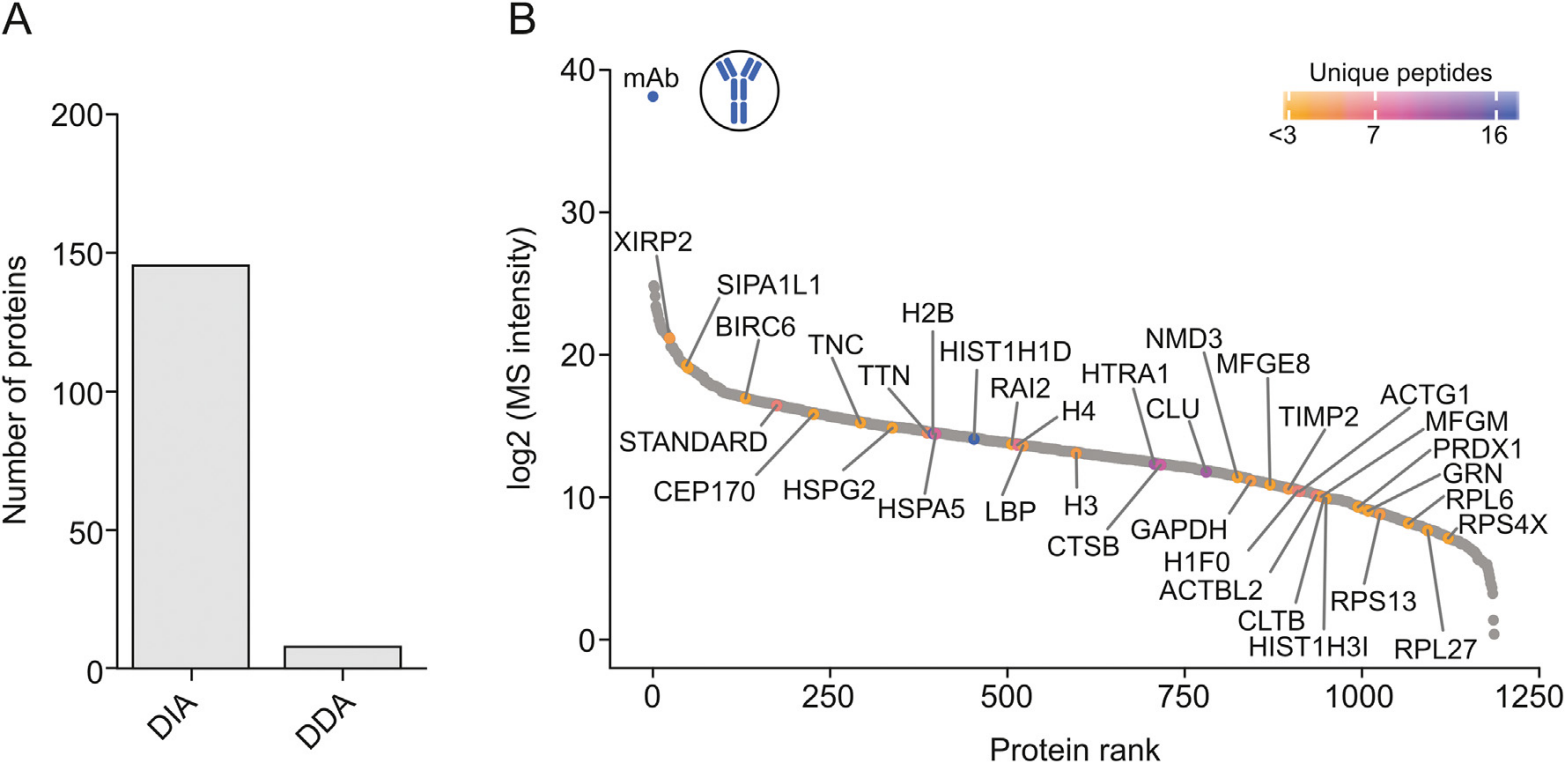
HCP identification and quantitation in protein A-purified IgG1 samples. (A) Number of identified proteins based on ≥2 unique peptides employing DDA- versus DIA-based analyses using the same chromatographic conditions. (B) Dynamic range plot of identified HCPs ranked according to their log2-transformed MS intensity using DIA-MS-based HCP detection. The color code indicates the number of unique peptides per quantified protein.

Here, the use of a DDA method, selecting the top five most intense ions for fragmentation, resulted in the identification of only eight host cell proteins with ≥2 unique peptides. The use of DIA employing variable isolation windows increased the number of identified proteins to 146, clearly showing the superiority of DIA over DDA (Fig. 2A). Furthermore, as shown in Fig. 2B, an unbiased quantitation of proteins over a broad abundance range was possible, highlighting the excellent sensitivity that can be achieved when using DIA-MS-based HCP detection. Using Hi3 quantitation, several HCPs that have previously been reported to be problematic were quantified at low amounts (below 5 ng/mg). In addition to immunogenic HCPs, such as clusterin (CLU) and histone H2B, proteases such as cathepsin B (CTSB) and HtrA serine peptidase 1 (HTRA1), which have been previously shown to affect product stability, were found [[19], [20], [21], [22]].

### 3.3 HCP profiling following altered bioprocessing

Improved knowledge about the effects of cell culture and downstream processing conditions on the HCP profile has led to tremendous improvements in recombinant protein production upon applying risk-based approaches [23]. However, as recently summarized by Gilgunn et al. [3], certain proteins that are difficult to remove can remain problematic, especially if they degrade the drug substance or are potentially immunogenic. Thus, commonly observed contaminants pose a great risk for product stability [15] and/or safety [24] and therefore, require close monitoring using highly sensitive analytical methods.

**Fig. 3:**
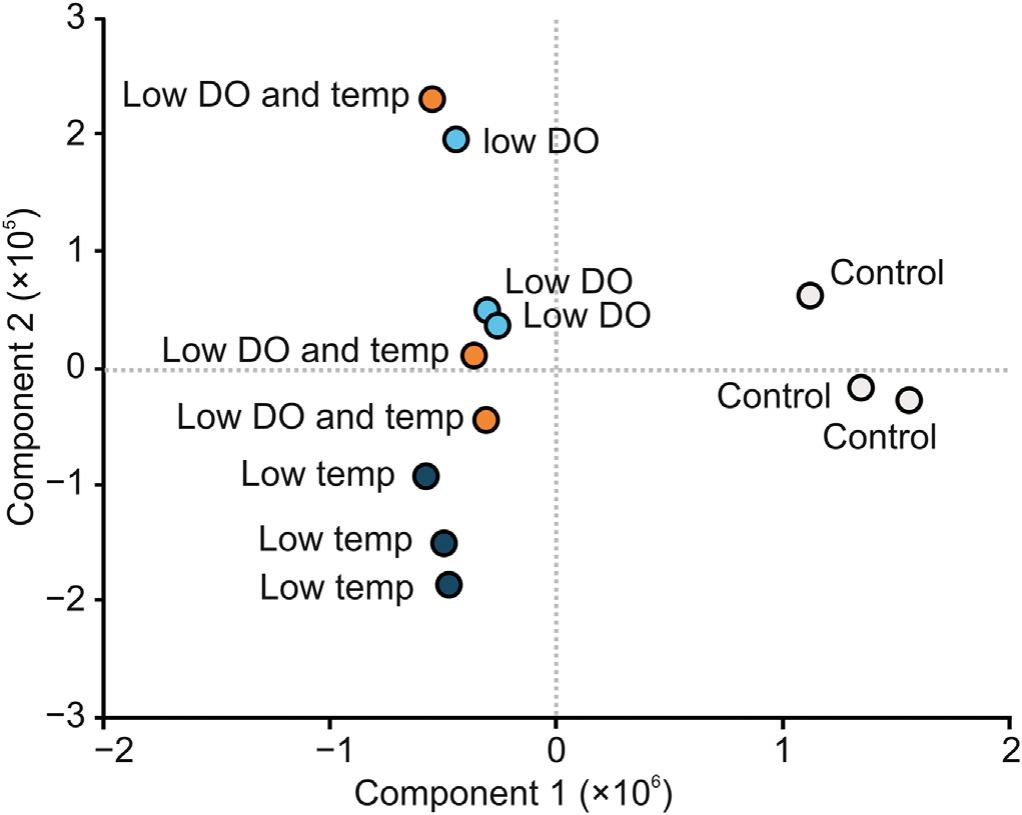
Principal component analysis (PCA) of HCPs detected in protein A-purified IgG1 samples obtained after altered bioprocessing conditions. Gray circles show the control samples, dark blue circles indicate samples obtained after lowering the cell culture temperature (from 37 °C to 32 °C), light blue circles show reduced DO concentration (from 40% to 20%), and orange circles indicate a combination of low temperature and low DO concentration. All samples were analyzed in triplicate.

To demonstrate the potential of the presented method, the HCP profile of CHO DP-12-derived IgG1 after altered bioprocessing conditions was analyzed. Statistical evaluation of the results using principal component analysis (PCA) showed that the HCP profiles of samples obtained after altering the cell culture conditions were different from those of the control samples generated using normal bioprocess conditions (Fig. 3). Using a one-sided *t*-test with a *P*-value threshold of 0.05, 33 HCPs were found to be significantly enriched when the cell culture bioprocessing parameters were changed (Table 1). In particular, lowering the temperature of the cell culture, a method commonly applied to increase recombinant protein production [25], was found to have a strong effect on the abundance of the detected proteins. Importantly, potentially immunogenic HCPs, such as H2B and CD8A, were found to be significantly enriched. While H2B was previously described to be problematic [3], an increase in CD8A could also be critical since it is involved in inducing innate immune responses, and therefore, might also cause potential unwanted immune reactions in patients. Additionally, when the effects of applied cell culture conditions were compared, HYOU1 and TRAF3 were found to be more abundant upon lowering the DO concentration. While TRAF3 is involved in TNF signaling, HYOU1 is related to hypoxia-induced stress responses in cells. Therefore, both proteins might be useful marker proteins for monitoring cellular stress levels during bioprocessing. Further investigations are required to determine whether these changes are critical to product quality. Nevertheless, these findings demonstrate the great potential of the developed method to monitor alterations in upstream processing. Using a DIA-based LC-MS/MS analysis of bioreactor-derived mAb samples enabled the detection and quantitation of relevant HCPs, thereby allowing for clear differentiation between the effects caused by the applied conditions.

**Table 1:** Significantly enriched HCPs detected in IgG1 samples obtained after altering the bioprocessing conditions (low temp = temperature reduced to 32 °C; low DO = DO level lowered to 20%). Shown in the table are the fold changes when comparing the altered conditions vs. control, as well as the protein concentrations in ng/mg (ng of HCP/mg of mAb). Significance was determined using a *t*-test with a *P*-value threshold of 0.05*.*

Accession	Gene name	Low DO		Low temp		Low DO & temp	
		Protein change	Conc. (ng/mg)	Fold change	Conc. (ng/mg)	Fold change	Conc. (ng/mg)
G3I9V7	APOBEC2			1.86	2.00		
G3IKK5	ART2B			5.05	20.61		
G3HAA4	ATOH7	2.57	10.35	1.92	5.99		
G3HR96	BLMH			4.50	13.08		
G3H9V1	C1ORF216	6.05	4.58	6.66	5.61	6.13	4.04
G3I2F3	CATSPER1			2.35	1.02		
G3HJJ9	CD8A	3.18	1.82	3.08	1.24		
G3IJZ9	CDK11B	5.85	1.61	6.07	1.31		
G3GYI4	DGKZ	1.65	1.43	1.82	1.52		
G3HC12	GATAD2B			3.63	4.63		
G3H2T4	H2B	3.98	1.40	4.04	1.49		
G3HDU1	HIST1H1D			5.54	1.14		
G3H7T9	HUWE1	4.97	1.58	5.00	1.37	3.58	1.34
G3I973	HYOU1	6.45	2.34	5.37	1.60	5.84	1.68
G3H3J9	KIF21B	5.06	11.42	5.17	12.59	4.79	10.77
G3GYH8	MAPK8IP1			4.42	6.67	4.87	8.93
G3HF29	MIER2	4.67	1.75	4.82	1.12	3.92	1.21
G3HJS7	MTIF2	5.26	17.92	4.86	16.03	3.96	16.08
G3HSA2	MYBBP1A	5.02	5.64	5.68	4.86		
G3IJF2	NUFIP2			3.02	2.19		
G3IE17	OLFR2W3			3.11	11.98		
G3ICS5	OLFR532	4.07	2.78	4.25	3.94	3.75	2.48
G3H4A3	PGPEP1	3.42	1.73	3.85	3.50	3.33	2.31
G3HT08	PLRG1	2.01	7.85	1.83	6.36		
G3GVR2	PTBP1			4.55	6.69		
G3I7Z7	RASA3	5.15	3.38	6.68	2.59	3.93	2.65
G3IIB1	SIAE			6.12	1.22		
G3H777	SLC25A5	3.12	2.44	3.07	2.32	2.18	1.80
G3HI05	SLC31A1	5.74	5.37	5.52	4.05	4.50	3.95
G3I8Z3	TBC1D20	3.97	1.22	4.44	1.16		
G3H369	TRAF3	8.30	7.92	6.28	6.71	6.40	6.32
G3HTF3	VPS18	4.12	1.40	4.50	1.41	4.53	2.24
G3HME8	ZBTB5	5.22	4.93	5.79	3.62	4.63	3.53

## 4 Conclusion

Previously presented methods for HCP analysis were either focused on automation, deep HCP coverage, robustness, or suitability for regulatory environments. Here, we described a novel strategy that combines all the aforementioned characteristics in a single, easy-to-implement method. Hence, the present study constitutes a workflow for HCP detection and quantitation of antibody drug products with great potential for applications in the biopharmaceutical industry. Automated sample preparation using commercially available magnetic beads enables high throughput by reducing the processing time while allowing for high reproducibility and robustness. Additionally, employing the same instrumental LC-MS setup that is routinely used for peptide mapping analysis of mAbs allows for the easy implementation of this method, demonstrating the high system flexibility of modern LC-MS instrumentation. The proposed method might also be used in multi-attribute monitoring workflows, although this requires further development. However, by using a DIA-based LC-MS method, the detection and quantitation of commonly observed problematic HCPs, namely, cathepsin B and HTRA1, were enabled. Furthermore, it was possible to monitor changes in the HCP profile upon alterations in the bioprocessing conditions. This demonstrates the suitability of the proposed method for monitoring CQAs that may impact drug efficacy and safety.

## Declaration of competing interest

J. Bones received funding to support undertaking this study as part of a funded collaboration between NIBRT and Thermo Fisher Scientific. G. Oliviero, C. Jakes, M.R. da Silva, and S. Carillo are or were employed on this collaborative project. Beyond this, the authors are not aware of any affiliations, memberships, funding or financial holdings that might perceive as affecting the objectivity of this article.
